# Novel compound heterozygous MYO15A splicing variants in autosomal recessive non-syndromic hearing loss

**DOI:** 10.1186/s12920-023-01777-4

**Published:** 2024-01-02

**Authors:** Kaifeng Zheng, Sheng Lin, Jian Gao, Shiguo Chen, Jindi Su, Zhiqiang Liu, Shan Duan

**Affiliations:** 1 Laboratory of Molecular Medicine, Institute of Maternal and Child Medicine, Affiliated Shenzhen Maternity & Child Healthcare Hospital, Southern Medical University, Shenzhen, China; 2 Shenzhen Health Development Research and Data Management Center, Shenzhen, China

**Keywords:** Non-syndromic hearing loss, MYO15A, Genotype, Splicing variant

## Abstract

**Background:**

Hereditary hearing loss is a highly heterogeneous disorder. This study aimed to identify the genetic cause of a Chinese family with autosomal recessive non-syndromic sensorineural hearing loss (ARNSHL).

**Methods:**

Clinical information and peripheral blood samples were collected from the proband and its parents. Two-step high-throughput next-generation sequencing on the Ion Torrent platform was applied to detect variants as follows. First, long-range PCR was performed to amplify all the regions of the GJB2, GJB3, SLC26A4, and MT-RNR1 genes, followed by next-generation sequencing. If no candidate pathogenetic variants were found, the targeted exon sequencing with AmpliSeq technology was employed to examine another 64 deafness-associated genes. Sanger sequencing was used to identify variants and the lineage co-segregation. The splicing of the MYO15A gene was assessed by in silico bioinformatics prediction and minigene assays.

**Results:**

Two candidate MYO15A gene (OMIM, #602,666) heterozygous splicing variants, NG_011634.2 (NM_016239.3): c.6177 + 1G > T and c.9690 + 1G > A, were identified in the proband, and these two variants were both annotated as pathogenic according to the American College of Medical Genetics and Genomics (ACMG) guidelines. Further bioinformatic analysis predicted that the c.6177 + 1G > T variant might cause exon skipping and that the c.9690 + 1G > A variant might activate a cryptic splicing donor site in the downstream intronic region. An in vitro minigene assay confirmed the above predictions.

**Conclusions:**

We identified a compound heterozygous splicing variant in the MYO15A gene in a Han Chinese family with ARNSHL. Our results broaden the spectrum of MYO15A variants, potentially benefiting the early diagnosis, prevention, and treatment of the disease.

## Background

Deafness is a prevalent neurosensory impairment globally, with an estimated incidence of 1–2 in 1000 newborns experiencing congenital or prelingual deafness. More than half of these cases are attributed to genetic factors [1, 2]. Non-syndromic hearing loss (NSHL), which accounts for approximately 70% of hereditary deafness cases without accompanying symptoms, is the most common form of hereditary deafness. Hereditary deafness can be categorized into autosomal dominant, autosomal recessive, mitochondrial, and X-linked patterns. Among these, autosomal recessive non-syndromic hearing loss (ARNSHL) is the prevailing hereditary pattern [3]. Currently, there have been reports of 105 genetic loci and 67 genes associated with hereditary deafness in ARNSHL (Hereditary Hearing Loss Homepage, http://hereditaryhearingloss.org), and the genes GJB2, SLC26A4, MYO15A, OTOF, and CDH23 are frequently implicated in ARNSHL [4].

The MYO15A gene, located on chromosome 17p11.2 (chr17:18,012,020 − 18,083,116; hg19 assembly), spans 71 kb of DNA and consists of 66 exons. It encodes three distinct isoform classes, one of which is 395 kDa (isoform 1), the largest member of the myosin superfamily of molecular motors [5]. The Myosin 15 protein is composed of four structural regions: (i) a 133-kDa N-terminal extension encoded by exon 2; (ii) the motor ATPase domain; (iii) two light chain binding IQ motifs (iv) a tail region containing two myosin-tail homology 4 (MyTH4) domains, two band 4.1/ezrin/radixin/moesin (FERM) domains, an Src-homology-3 (SH3) domain and a C-terminal class I PDZ-ligand domain [6, 7].

Variants in the MYO15A gene have been identified as a causative factor for sensorineural hearing loss in humans, specifically in cases of autosomal recessive 3 (OMIM, #600,316). In a genome-wide disequilibrium study conducted in 1995, an association between MYO15A and deafness was first discovered in an isolated village in Indonesia, where approximately 2% of the population was affected by hearing loss [8, 9]. Clinically, variants in MYO15A have been found to result in congenital severe to profound hearing loss across all frequencies, although some patients may retain residual hearing at lower frequencies. Further investigations using myosin XVa-deficient mice have confirmed the role of MYO15A variants in the development of deafness.

The findings from studies conducted on myosin XVa-deficient mice have demonstrated that variants in the MYO15A gene can lead to deafness through two distinct mechanisms: the failure to develop mechanosensory hair bundles and the inability to sustain mature stereocilia. This indicates that myosin XVa plays a crucial role in both the initial formation and long-term maintenance of stereocilia [10].

In the present study, a compound heterozygous variant involving NG_011634.2 (NM_016239.3): c.6177 + 1G > T and c.9690 + 1G > A, situated within the MYO15A gene, has been identified in a Chinese family with ARNSHL using targeted next-generation sequencing. These variants have been predicted by three in silico tools to disrupt the native splicing donor site of the MYO15A gene, leading to exon skipping or activation of cryptic splice sites, ultimately resulting in deleterious effects on protein functionality. Subsequent in vitro experimentation utilizing a minigene splicing assay has confirmed this prediction. Based on the guidelines provided by the American College of Medical Genetics and Genomics (ACMG), it can be inferred that this compound heterozygous variant is responsible for the occurrence of deafness within this particular family.

## Methods

### Subjects and clinical evaluation

A Chinese family who was impacted by ARNSHL was recruited from the Laboratory of Molecular Medicine, Shenzhen Health Development Research Center. Peripheral blood samples were collected from all members of the family. The proband underwent a comprehensive clinical evaluation, which included otologic examinations, audiology testing, and temporal bone computer-assisted tomography (CT). The hearing level was assessed at frequencies of 500, 1000, 2000, and 4000 Hz using the pure tone average (PTA) method and categorized as normal (≤ 25 dB HL), mild (26 ~ 40 dB HL), moderate (41 ~ 60 dB HL), severe (61 ~ 80 dB HL), and profound (≥ 80 dB HL) deafness. The utilization of a cohort consisting of 200 Chinese Han individuals with normal hearing and devoid of any other known diseases as the control group. The study was reviewed and approved by the Ethics Committee of Shenzhen Health Development Research Center (Approval no. 2019-012). Informed consent was obtained from each participant or, in the case of a minor, from the parents. All procedures involving human participants were done following the ethical standards of the institutional or national research committee and with the 1964 Helsinki Declaration and its later amendments or comparable ethical standards.

### Library construction and targeted next-generation sequencing

Genomic DNA was extracted from whole blood using the UPure Blood DNA Extraction Kit (Bio-Base Technologies, China). DNA concentrations were estimated using the Qubit™ dsDNA HS Assay Kit (Thermo Fisher Scientific, USA) on the Qubit® 2.0. Fluorometer (Thermo Fisher Scientific, USA).

Previous studies have reported the identification of variants in the GJB2, SLC26A4, MT-RNR1, and GJB3 genes in over 30% of Chinese individuals with hearing impairment [11]. To enhance efficiency and reduce costs associated with detection, a two-step testing approach was employed. Initially, the four commonly implicated deafness genes (GJB2, GJB3, SLC26A4, and MT-RNR1) were subjected to prescreening in the proband. If no suspected pathogenic variant was detected, the proband underwent further screening using targeted next-generation sequencing (NGS) of 64 known genes associated with deafness.

**Table 1:** Primer sequences for the long-range PCR of the four common deafness genes

Gene	Forward	Primer sequence	Reverse	Primer sequence
MT-RNR1	12SrRNA-F	GTTTAGACGGGCTCACATCACC	12SrRNA-R	GGTTTGGCTAAGGTTGTCTGGT
GJB2	GJB2-F	ATGTCCAGCCAACTCAGCATTCAC	GJB2-R	ACAAGCCGACTCAGCACATCTCTC
GJB3	GJB3-F	GGCTCCCAACTCAGTGCTCATTCTC	GJB3-R	TGATGACCCCACCTGTCTTTGCTG
SLC26A4	SLC26A4-1 F	AAATCGTGAACTCTCTGTATCCTCC	SLC26A4-1R	GCTGACCTCAAACATCTACCTTATC
SLC26A4	SLC26A4-2 F	CAGATGTAGATGCCTTCAGTCCAGT	SLC26A4-2R	TCCAGAGCAGCCTTTACTTCATTAG
SLC26A4	SLC26A4-3 F	TCTAATGAAGTAAAGGCTGCTCTGG	SLC26A4-3R	CGGCTGTTTTCATTATCCTTGTCT
SLC26A4	SLC26A4-4 F	AGGTCCTTCATTCATCTCTGTCAC	SLC26A4-4R	GGATTTCAGTTCAGGCACTTAGAC
SLC26A4	SLC26A4-5 F	ATGTCCATTCTCCTGATGTTACCTC	SLC26A4-5R	TGGGCAACCCTACCTCCAATAC
SLC26A4	SLC26A4-6 F	TTACTGGGGTCCAATCAGGAATAG	SLC26A4-6R	GCTTTGCTCTCCTCATAACAACATC
SLC26A4	SLC26A4-7 F	ATGGAAATGTGTATCTTGACAGTGG	SLC26A4-7R	GCCTCACAAGAATGTTCATAGTAGC

To amplify the whole sequences of the GJB2, SLC26A4, MT-RNR1, and GJB3 genes, long-range PCR (LR-PCR) was conducted. The primer pairs for LR-PCR were designed using the Primer5 program, and their details are presented in Table 1. The LR-PCRs were performed in a 25 µL volume, with approximately 25 ng of DNA serving as the template in a separate reaction. The final concentrations of the components were as follows: 1× Tks Gflex buffer (TaKaRa Bio Inc., Japan), 0.025 U/µL Tks Gflex DNA polymerase (TaKaRa Bio Inc., Japan), and 0.2 µM of both the forward and reverse primers. The thermal-cycling procedure consisted of an initial denaturation step at 94 °C, followed by 30 cycles of amplification at 94 °C for 1 min, 65 °C for 30 s, and 68 °C for 10 min. The final elongation step lasted for 30 min at 68 °C, followed by a hold at 10 °C. The resulting PCR products were then purified and pooled in equal molar ratios. A total of 1 µg of the pooled amplicons was utilized to generate the sequencing library using the Ion Xpress Plus Fragment Library Kit (Thermo Fisher Scientific, USA) according to the protocol provided by Ion Torrent (Thermo Fisher Scientific, USA). Subsequently, the library was sequenced using the Ion Torrent Proton sequencer (Thermo Fisher Scientific, USA).

**Fig. 1:**
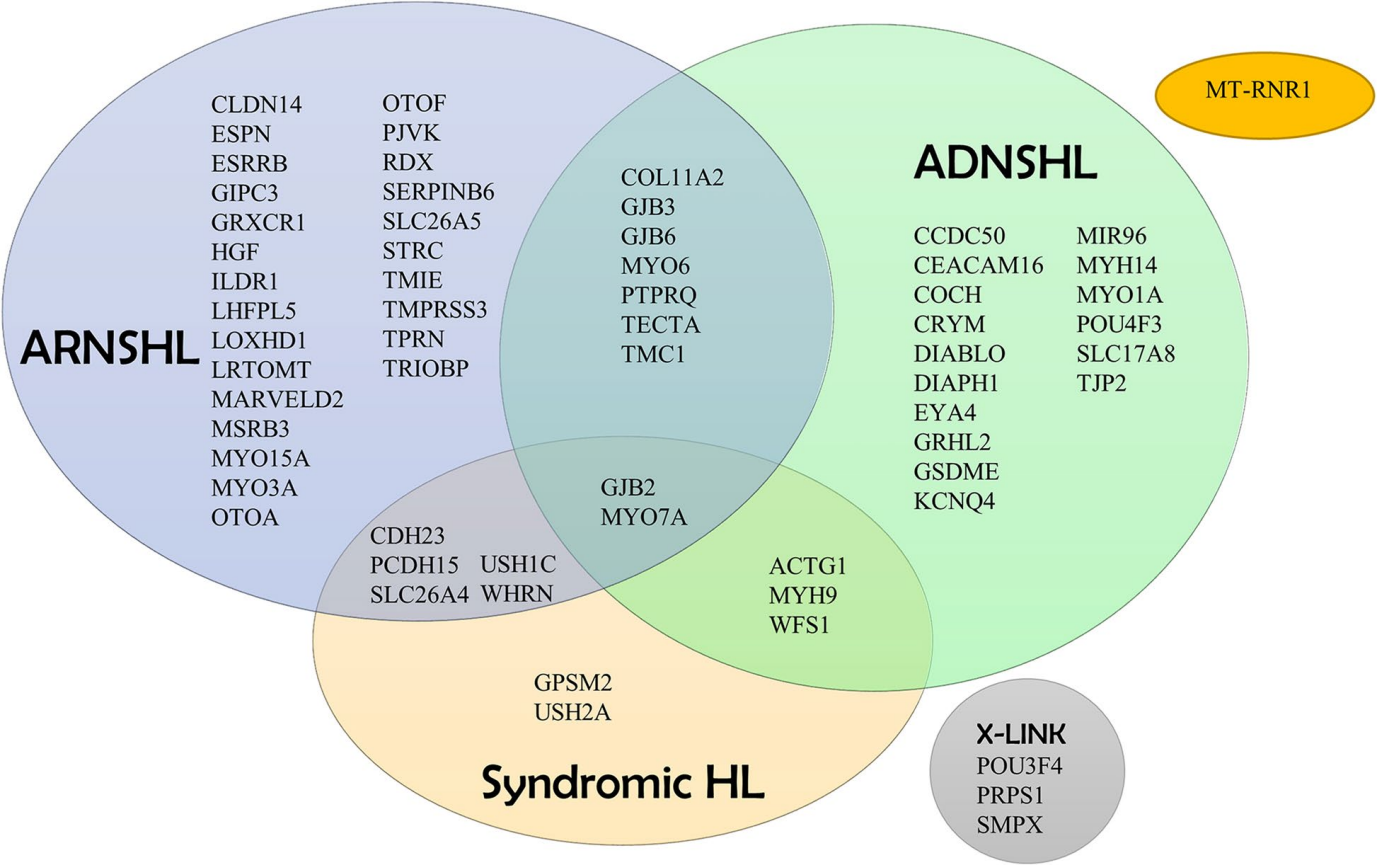
Genes included in the modified Hearing Loss Research Panel for NGS library construction

To enhance the amplification of the entire exon and exon-flanking region of 64 genes associated with hearing loss, a predesigned Ion AmpliSeq community contributed panel (Ion AmpliSeq™ Hearing Loss Research Panel v1 [12], Thermo Fisher Scientific, USA) was utilized with minor adjustments (supplemented with several missing gaps and MT-RNR1). This panel consists of 64 frequently identified genes linked to ARNSHL, ADNSHL, X-linked hearing loss, and even certain syndromic hearing loss conditions, as depicted in Fig. 1. The designed gene panel ensured a comprehensive coverage of the targeted region, encompassing 95.74% of the sequence spanning 294.81 Kb. The construction of libraries and subsequent next-generation sequencing were carried out on an Ion Torrent Proton machine following the manufacturer’s protocols.

### NGS data analysis

The data analysis was conducted using the Ion Torrent Suite v4.4, with variant calling performed by the Variant Caller plugin v4.4. Subsequently, single nucleotide variants (SNVs) and small insertion/deletions (INDELs) were annotated using ANNOVAR. The alignment of reads was visualized using the Integrative Genome Viewer (IGV) software. An in-house script was employed to evaluate the pathogenicity of each variation based on ANNOVAR annotation, following the guidelines established by the American College of Medical Genetics and Genomics (ACMG). Consequently, all variations were classified into five categories: pathogenic, likely pathogenic, variant of uncertain significance (VUS), likely benign, and benign. Only variants classified as pathogenic and likely pathogenic were deemed deleterious and confirmed through Sanger sequencing.

### Splice-site prediction analysis

In the case of variants potentially impacting mRNA splicing, predictive analysis was conducted using Human Splicing Finder 3.1 [13], NetGene 2 service [14], and NNSPLICE 0.9 [15]. We consider potentially relevant those motifs (acceptor or donor sites) that are disrupted or created as a consequence of a specific variant with a score variation (difference between the reference wild type and mutant motif’s strengths) > 20%, and in case of creating a new motif when it also reaches a score > 80%. NetGene 2 and NNSPLICE present scores of 0–1 for the predicted site; the higher the score, the more likely a variant is a splicing site.

### Minigene splicing assay

In vitro, minigene assays were performed using the preconstructed pET01 Exontrap vector (MoBiTec, Goettingen, Germany), which encodes a 5′ and 3′ exon separated by a multiple cloning site. Gene-specific primers were designed in the 3′ and 5′ flanking intron regions of MYO15A exons 28 and 59, respectively, and these primers contained either BamHI-HF or XhoI restriction enzyme sites. The primer sequences used were as follows: Ex28-F: (BamHI-HF) 5’-CCGCTCGAGGGGCCCAGGCAGATCAGAG-3’ (the underline indicates the restriction enzyme site, same as below). Ex28-R: (XhoI) 5’-CGCGGATCCAAGACACAGGGCAGGCACAT-3’, Ex59-F: (BamHI-HF) 5’-CCGCTCGAGCAAGGGCTGTCCCAGATCC-3’, Ex59-R: (XhoI) 5’-CGCGGATCCCATGCCAGTTGGGCTTGAC-3’. MYO15A exon 28 and 59, along with their respective flanking introns, were amplified by PCR using the gene-specific primers and gDNA from the proband as the template. Following digestion with XhoI and BamHI-HF (NEB, USA), the PCR fragment was ligated into the pET01 vector for transformation into DH5α competent cells (TIANGEN, China). Colonies were selected, cultured, and plasmid DNA was extracted using the Endo-free Plasmid Mini Kit II (Omega, USA). We generated wild-type and mutant minigene plasmids for the two variants c.6177 + 1G > T and c.9690 + 1G > A, respectively, and designated them as follows: pET01-Ex28-WT (c.6177 + 1G), pET01-Ex28-MT (c.6177 + 1T), pET01-Ex59-WT (c.9690 + 1G), and pET01-Ex59-MT (c.9690 + 1 A). Following sequence confirmation, the four minigene plasmids, and the control pET01 plasmid were introduced into HEK293 cells (human embryonic kidney, ATCC, United States) via transfection. Total RNA was subsequently isolated 48 h after transfection using the RNeasy Mini Kit (QIAGEN, Germany). Utilizing a primer that specifically targets the 3′ native exon of the pET01 vector, complementary DNA (cDNA) was synthesized employing RNA SuperScript™ III Reverse Transcriptase (Thermo Fisher Scientific, USA). Subsequently, the products obtained from PCR amplification were visualized on a 2% agarose gel, extracted, and subjected to sequencing.

## Results

### Clinical manifestations of the proband

**Fig. 2:**
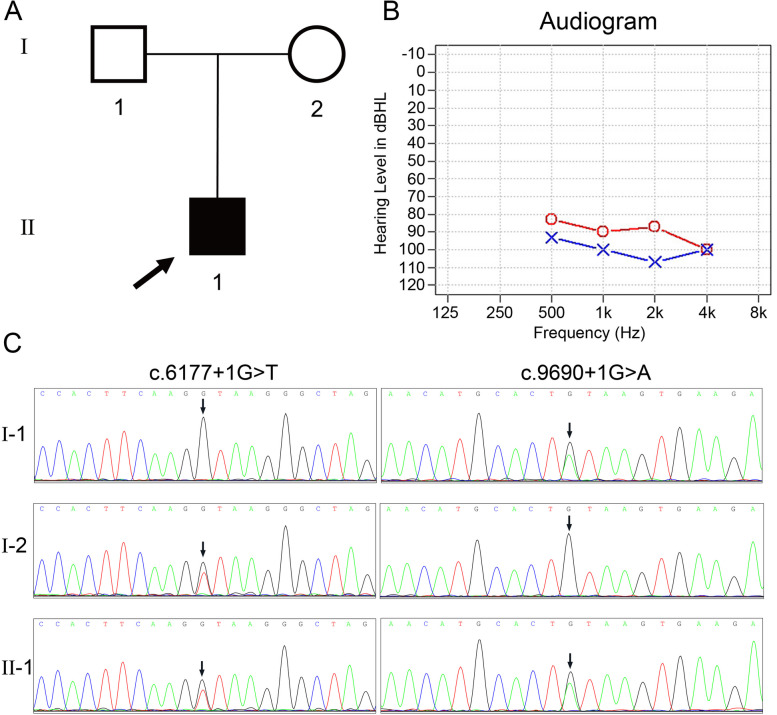
Combined figure. **A** Pedigree of the family with ARNSHL. The arrow indicates the proband, black represents deafness. **B** Auditory steady state response (ASSR) audiogram of the proband. **C** Sanger sequencing results of the c.6177 + 1G > T and c.9690 + 1G > A mutations in all family members. Arrows indicate the position of the mutations. The results showed that the compound heterozygous mutation cosegregated with the phenotype

The proband, identified as II-1 in Fig. 2a, exhibits congenital bilateral profound deafness and is an eight-year-old male. The hearing test was not administered immediately after birth, and at the age of 10 months, he displayed no response to sound. Subsequently, the transient evoked otoacoustic emission (TEOAE) test yielded negative results, and the ASSR audiogram (Fig. 2b) indicated profound hearing loss in both ears. The temporal bone computed tomography (CT) scan and magnetic resonance imaging (MRI) did not reveal any abnormalities. The proband was clinically diagnosed with profound non-syndromic sensorineural deafness, while his parents (I-1 and I-2, Fig. 2a) exhibited normal hearing. No familial occurrence of congenital hearing loss was documented, and potential environmental factors were ruled out.

### Targeted NGS of 64 deafness genes identified the pathogenic candidates

No pathogenic variants were identified in the proband through prescreening of GJB2, GJB3, SLC26A4, and MT-RNR1. Subsequently, the proband underwent targeted NGS of 64 deafness genes. Following data analysis and pathogenicity assessment based on the ACMG guidelines, only two variants were considered as potential pathogenic candidates. These two candidates, namely the compound heterozygous variants c.6177 + 1G > T and c.9690 + 1G > A of the MYO15A gene, were consistent with the recessive inheritance pattern observed in the family. Sanger sequencing confirmed that these variants were heterozygous in the proband’s parents (Fig. 2c). The variant c.6177 + 1G > T, which was absent in a cohort of 200 Chinese Han individuals with normal hearing, was found to potentially result in abnormal splicing of the mRNA. Notably, this specific variant has not been documented in previous studies, the 1000-Genomes Project, or the NHLBI Exome Sequencing Project. Many variants were observed in the proband within the detectable range of our NGS panel. Following the ACMG guidelines, it was determined that two variants in the MYO15A gene were pathogenic (class 1), meeting the PVS1, PM2, and PP3 criteria. Additionally, one variant located in the USH2A gene was classified as VUS, while the remaining variants were deemed benign or likely benign.

### In silico prediction the pathogenicity of candidate variants

**Fig. 3:**
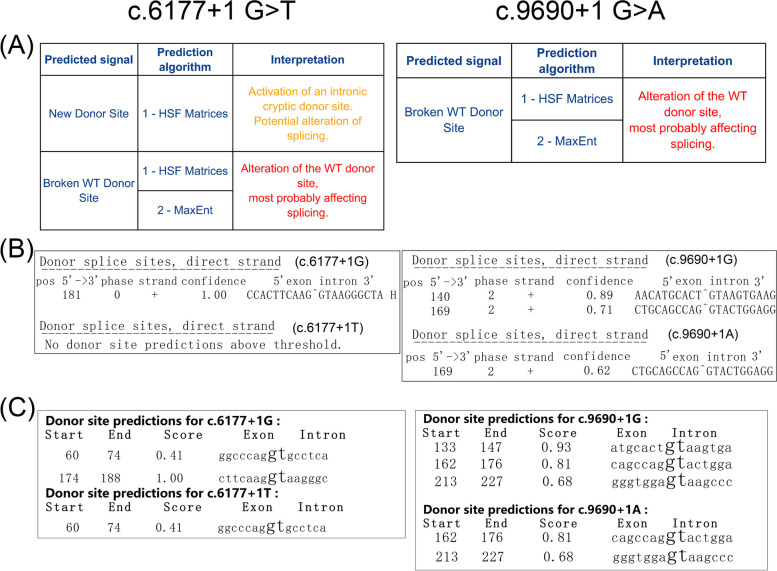
Results of in silico predictions. **A**-**C** rows represent the analysis results of three in silico prediction tools, respectively. **A** Human Splicing Finder3.1. **B** NetGene 2 service. **C** NNSPLICE 0.9. According to each tool, the result of c.6177 + 1G > T mutation is in the left side and the result of c.9690 + 1G > A mutation is in the right side

The pathogenicity of the variants was assessed using three silico prediction tools: HSF, NetGene2, and NNSPLICE 0.9. First, the HSF program indicated that both variants, c.6177 + 1G > T and c.9690 + 1G > A, were likely to disrupt the original splicing donor site, leading to the loss of normal splicing function (Fig. 3a). Additionally, the neural network of NetGene2 service and NNSPLICE 0.9 employed a rule-based system algorithm to predict donor and acceptor sites in human genes. In the case of c.6177 + 1G > T and c.9690 + 1G > A, NetGene2 accurately identified the donor site in the wild-type sequence with a score of 1.00 and 0.89, respectively, but failed to detect the mutant sequence (Fig. 3b). For the variants c.6177 + 1G > T and c.9690 + 1G > A, NNSPLICE 0.9 accurately predicted normal splicing by the wild type donor site with scores of 1.00 and 0.93, respectively. However, the mutant splice site was predicted to be abolished (Fig. 3c). The results obtained from these three in silico prediction tools strongly indicate that these two variants may disrupt normal splicing of the MYO15A gene. Furthermore, both the NetGene2 service and NNSPLICE 0.9 predicted the presence of a cryptic donor downstream of the position c.9690 + 1 in intron 59 of the MYO15A gene, while the HSF program did not.

### Variants confirmed to be pathogenic by splicing experiment

**Fig. 4:**
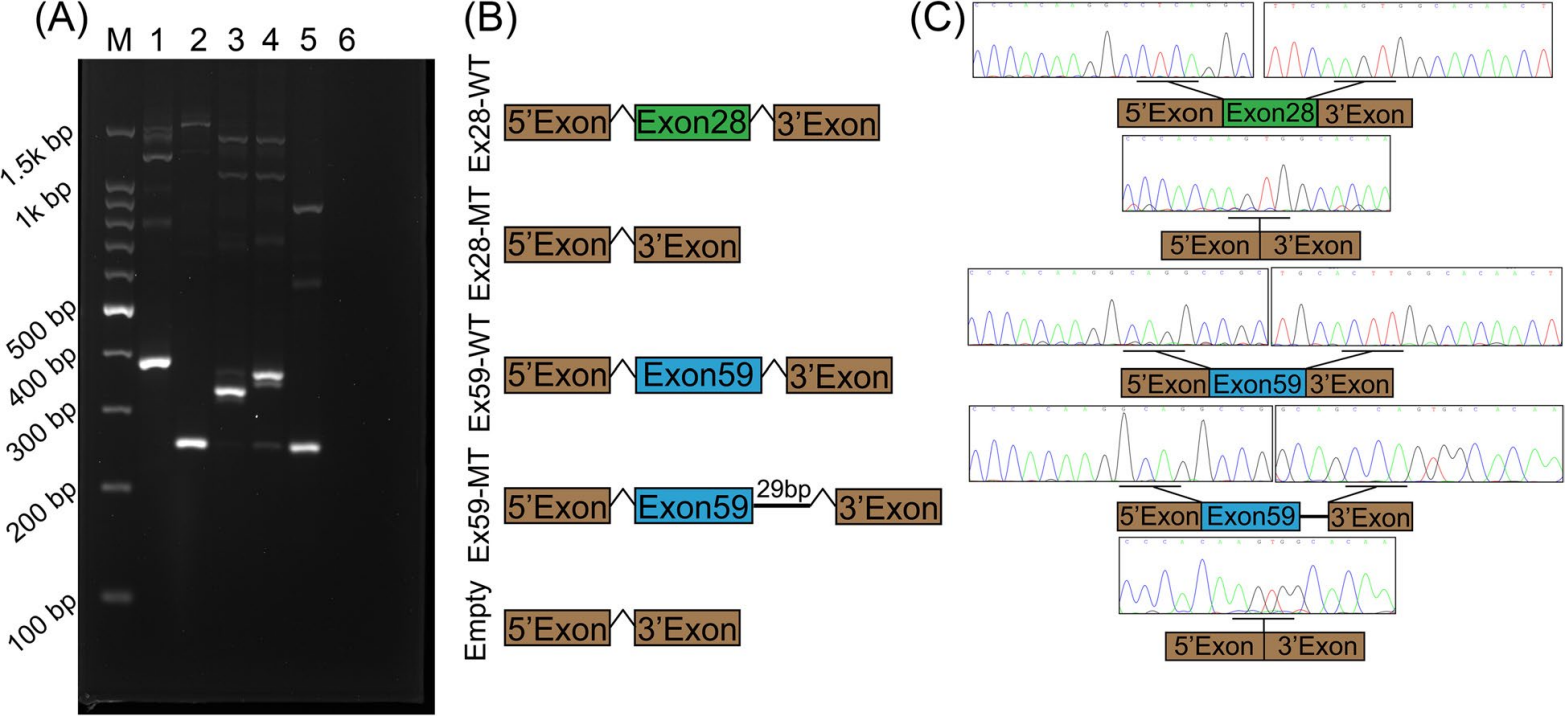
Minigene splicing assay of c.6177 + 1G > T and c.9690 + 1G > A in MYO15A using pET01 vector. **A** The RT-PCR results of minigene splicing assay are shown. M: DNA marker; 1–5 represent RT-PCR products of HEK293 cells transfected with the following five plasmids: (1) pET01-Ex28-WT plasmid; (2) pET01-Ex28-MT plasmid; (3) pET01-Ex59-WT plasmid; (4) pET01-Ex59-MT plasmid; (5) empty pET01 vector; (6) no template negative control. **B** Graphical illustration of the expected splicing products. **C** Representative Sanger sequencing results of the splicing products in HEK293 cells transfected with different plasmids

Total RNA was isolated from the transfected HEK293 cells, and its concentration and quality were evaluated through absorbance measurements at 260 and 280 nm. Reverse transcription (RT) was performed to synthesize complementary DNA (cDNA), which was subsequently amplified using specific primers. Visualization of the splicing products demonstrated that cells transfected with the pET01-Ex28-WT plasmid exhibited the anticipated 377-bp band containing exon 28 (Fig. 4a), while cells transfected with the pET01-Ex28-MT plasmid only produced a 246-bp product, similar to the empty pET01 plasmid (Fig. 4b),. This outcome was in agreement with the in silico prediction. Cells transfected with the pET01-Ex59-WT plasmid exhibited the anticipated 324-bp band containing exon 59, whereas cells transfected with the pET01-Ex59-MT plasmid displayed a longer product of 353 bp. This outcome indicated that the variant c.9690 + 1G > A disrupted the original donor site of intron 59, activated a novel donor site, and consequently led to the partial retention of intron 59 (Fig. 4b). These findings were in accordance with the predictions made by NetGene2 and NNSPLICE 0.9. The products were purified and the aforementioned results were validated through Sanger sequencing (Fig. 4c).

Based on the findings from the minigene splicing assay, we conducted an analysis of the mRNA sequences to determine whether the two variants under investigation result in frameshifts and the introduction of a premature stop codon. This analysis was performed using the Open Reading Frame Finder tool (https://www.ncbi.nlm.nih.gov/orffinder/). The results revealed that the variant c.6177 + 1G > T, which leads to the skipping of exon 28, would cause a frameshift in the MYO15A cDNA ORF and the introduction of a premature stop codon (NP_057323.3: p.Thr2017Leufs*62). Similarly, the variant c.9690 + 1G > A, which results in the partial retention of intron 59, would also cause a frameshift in the MYO15A cDNA ORF and the introduction of a premature stop codon (NP_057323.3: p.Ile3231Valfs*25). Consequently, these splicing variants are expected to give rise to an abnormal protein.

## Discussion

MYO15A variants have been identified as a prominent etiology of autosomal recessive non-syndromic hearing loss (ARNSHL) on a global scale. Pathogenic variants within the MYO15A gene have been found to result in congenital severe to profound hearing loss across all frequencies. Multiple studies have demonstrated that the most common pathogenic variants associated with hearing loss are located within the motor, FERM, and MyTH4 domains of the MYO15A gene [16, 17].

Notably, the prevalence of MYO15A-related deafness exhibits variability based on the ethnicity and country of the study population. For instance, a relatively low frequency of 0.89% (10/1120) was reported in Japan [18], while the Middle and South Asian areas reported a frequency of 3.3% [17]. In the Chinese ethnic population, the frequency was 4% (5/125) before excluding common variants [19]. In a Pakistani population with recessively inherited severe to profound hearing loss, the frequency was 5% [20], and in an Iranian population, it was 5.71% before excluding GJB2 variants [21]. Therefore, it is crucial to comprehensively analyze the variant spectrum of the MYO15A gene in each country for the diagnosis and prevention of hearing loss.

In this study, two potential MYO15A gene heterozygous splicing variants, namely c.6177 + 1G > T and c.9690 + 1G > A, the heterozygous nature of these variants were confirmed in the proband’s parents, consistent with an autosomal recessive inheritance pattern. Both variants were classified as pathogenic based on the guidelines set forth by the American College of Medical Genetics and Genomics (ACMG), and three in silico prediction tools predicted their potential to induce aberrant splicing of the mRNA. Based on previous studies and data from the HGMD database, a total of 360 pathogenic variants in the MYO15A gene have been identified. Among these variants, missense mutations outside the exon region were found to be the most prevalent, followed by nonsense mutation and splice site mutation [22]. Notably, two specific single splice site variants, namely c.6177 + 1G > T and c.9690 + 1G > A, were discovered in the Chinese population and have been previously reported. In this paper, a variant known as c.6177 + 1G > T was observed in two Chinese Han families, where it formed a compound heterozygous variant in conjunction with the variant c.3658_3662del [23] and c.10257_10259delCTT [24], respectively. The variant c.9690 + 1G > A was initially identified as a homozygous pathogenic variant in a Chinese Uyghur family in 2015 [25]. Additionally, four cases of this variant were observed in a Chinese Han family, consisting of one homozygous mutation and three cases of compound heterozygous mutation (in conjunction with one of the following variants: c.3971 C > A, c.4011insA, and c.10257_10259delCTT) [3, 24]. However, the novel pathogenic model involving the compound heterozygous mutation formed by these two variants, as discovered in this study, has not yet been reported.

No comprehensive experimental investigations have been undertaken to ascertain the impact of these two variants on the molecular function of MYO15A. The minigene strategy was utilized to confirm the impacts of the two variants. The c.6177 + 1G > T variant causing exon 28 skipping, and the c.9690 + 1G > A variant resulting in partial retention of intron 59. These findings align with the predictions made by the aforementioned tools (NetGene2 Service and NNSPLICE 0.9), which identified a potential donor splice site at 28 bp (c.9690 + 29) downstream of the c.9690 + 1G > A variant.

Based on the findings obtained from the minigene assay, it can be deduced that the splice site variants c.6177 + 1G > T and c.9690 + 1G > A would induce a frameshift and the occurrence of a premature stop codon. This occurrence has the potential to initiate the process of nonsense-mediated decay (NMD) or produce a truncated protein. The activation of NMD would subsequently lead to the manifestation of the disease, as it is associated with the functional haploinsufficiency of the MYO15A gene. Conversely, the absence of NMD would result in the production of a truncated protein. The c.6177 + 1G > T variant, situated within the MyTH4 domain of myosin XVA, is anticipated to cause a substantial reduction in functional protein. Furthermore, the c.9690 + 1G > A variant, located in the junctional region between the posterior MyTH4 domain and the FERM domain of myosin XVA, is expected to yield a truncated protein lacking a segment of the second FERM domain and the PDZ-ligand in the tail region of myosin XVA. This specific region plays a critical role in the normal operation of the stereocilia tips. Consequently, these two variants may also contribute to the pathogenesis of the disease.

Including additional variants of the MYO15A gene in the HL database is invaluable and significant in enhancing our comprehension of recessive hearing loss. Indeed, this highly depends on the specific family and patient [26, 27]. It is also helpful to identify genotype - phenotype correlations and future clinical trials [28].The de novo compound heterozygous mutations identified in this research broaden the spectrum of variants in the MYO15A gene and provide an essential basis for clinical diagnosis and guidance for the parents of affected children regarding reproduction decisions. Genetic counseling is imperative for families affected by this condition, as there is currently no available therapy. Furthermore, parenteral genetic screening/diagnosis and newborn screening are the best strategies for managing this disease [29–31].

By broadening the range of variants of genes, including MYO15A linked to hereditary hearing loss(HL), and employing highly precise sensitive methodologies, such as NGS-based NIPT [29], NGS-based pre-implantation genetic testing (PGT-A, PGT-M) [30], it would effectively avoid the occurrence of children with ARNSHL through pregnant women screening or routine-based embryo screening.

Additionally, Cochlear implantation (CI) is the only effective therapeutic strategy for individuals with severe sensorineural hearing loss (SNHL). Meanwhile, the influence of various genes like MYO15A associated with deafness on the outcomes of Cochlear implantation has been identified [32]. As a result, genetic screening and appropriate genetic counseling for deaf individuals are of utmost importance to determine the molecular cause of deafness and enhance the effectiveness of CI.

In addition, it is essential to acknowledge the limitations of our study. Firstly, the presence of the variant was only observed within a single family. To address this limitation, future investigations should consider incorporating a larger sample size to enhance the understanding of the variant’s pathogenicity. Secondly, conducting comprehensive functional research is necessary to evaluate the potential pathogenicity of the variants of the MYO15A gene.

## Conclusions

In conclusion, taking into account the phenotype, clinical diagnosis, gene detection, and experimental research findings, it is reasonable to ascribe the pathogenic etiology of this ARNSHL family to the compound heterozygous variants c.6177 + 1G > T and c.9690 + 1G > A in the MYO15A gene. Individuals with hearing impairment resulting from a variant in the MYO15A gene typically derive advantages from Cochlear transplantation. Additionally, other family members harboring these variants can undergo prenatal testing as a preventive measure against the occurrence of offspring with the same disorder. Moreover, our study provides substantial evidence that supports the pathogenic nature of the c.6177 + 1G > T and c.9690 + 1G > A variants, thereby broadening the spectrum of pathogenic variants associated with the MYO15A gene.

## Data Availability

The data supporting the findings of this study are available within the article and its supplementary materials and from the corresponding author, Shan Duan, upon reasonable request. The compound heterozygous variant c.6177 + 1G > T and c.9690 + 1G > A was submitted to ClinVar database (https://www.ncbi.nlm.nih.gov/clinvar/). The submission number was SCV004171060 and SCV004171061.

## Abbreviations

NGS: Next-generation sequencing
ARNSHL: Autosomal recessive non-syndromic sensorineural hearing loss
ADNSHL: Autosomal dominant non-syndromic sensorineural hearing loss
ACMG: American college of medical genetics and genomics
ASSR: Auditory steady-state response
TEOAE: Transient evoked otoacoustic emission
ORF: Open Reading Frame
NMD: Nonsense-mediated decay
